# Maternal prothrombin time as an independent predictor of neonatal sepsis: A retrospective case–control study

**DOI:** 10.1371/journal.pone.0345672

**Published:** 2026-03-20

**Authors:** Gökçenur Karakelleoğlu, Elif Ceren Nur Kırımlı Yanık, Şenol Bozdağ

**Affiliations:** 1 Department of Obstetrics and Gynecology, Istanbul Okan University Faculty of Medicine, Tuzla, Istanbul, Türkiye; 2 Department of Neonatology, Istanbul Okan University Faculty of Medicine, Tuzla, Istanbul, Türkiye; Haman Tis Research Institute, IRAN, ISLAMIC REPUBLIC OF

## Abstract

**Objective:**

To evaluate the diagnostic performance of maternal hemostatic and inflammatory markers in predicting neonatal sepsis.

**Methods:**

This retrospective case–control study included 176 newborns, of whom 37 were diagnosed with neonatal sepsis and 139 were healthy controls. Maternal blood samples collected at delivery were analyzed for white blood cell count (WBC), neutrophil-to-lymphocyte ratio (NLR), platelet count (PLT), prothrombin time (PT), activated partial thromboplastin time (aPTT), international normalized ratio (INR), and systemic inflammatory indices (SII, SIRI, PIV). Group comparisons were performed using the Mann-Whitney U and Chi-square tests. Predictive markers were assessed using multivariate logistic regression and ROC curve analysis.

**Results:**

Maternal PT was significantly higher in the sepsis group (p < 0.001), and multivariate logistic regression identified PT as the only independent predictor of neonatal sepsis (OR: 1.79; 95% CI: 1.39–2.31; p < 0.001). ROC analysis showed PT had the highest diagnostic accuracy (AUC: 0.909). The optimal cut-off value for PT was 10.25 seconds, yielding a sensitivity of 89.2% and specificity of 68.3%.

**Conclusion:**

Maternal PT demonstrates excellent diagnostic accuracy in predicting neonatal sepsis. As one of the first studies to apply logistic regression and ROC analysis solely to maternal hemostatic and inflammatory markers, our findings suggest that maternal PT could serve as a valuable, easily obtainable biomarker for early neonatal sepsis risk stratification.

## Introduction

Neonatal sepsis remains a significant cause of morbidity and mortality worldwide, particularly in preterm infants and those with low birth weight [1]. Timely diagnosis is critical but challenging due to nonspecific clinical signs and delayed laboratory confirmation. Although C-reactive protein (CRP) and procalcitonin (PCT) are widely used, their diagnostic accuracy is limited, especially for early detection [2].

Recently, maternal predictors have received increasing attention. For instance, a large retrospective cohort found that maternal leukocytosis and intrapartum fever are independent risk factors for early-onset neonatal sepsis (EOS), with maternal WBC > 16.75 × 10⁹/L showing an area under the curve (AUC) value of 0.82 for EOS prediction [3]. However, most studies focus on inflammatory markers—leukocyte count, CRP—but lack evaluation of maternal coagulation profiles.

Coagulation derangements are known to accompany neonatal sepsis through inflammatory-thrombotic cross-talk [4], and abnormalities in neonatal hemostasis, including prothrombin time,has been reported in septic infants [5,6]. Maternal PT is less studied; a Turkish perinatal cohort associated maternal PT > 14 s with neonatal morbidity [7], while a multicenter analysis suggested maternal PT > 16 s may predict 30-day mortality in septic neonates [8].

Furthermore, maternal inflammatory indices—such as SII, SIRI, and PIV—are emerging predictors in obstetrics (e.g., fetal growth restriction, preeclampsia, pregnancy loss) [9–12]. Yet their role in neonatal sepsis prediction is untested.

Intrauterine infection and inflammation, such as chorioamnionitis, are known to trigger a maternal systemic inflammatory response that may extend to the fetal compartment through placental signaling pathways [12]. This shared maternal–fetal inflammatory milieu has been implicated in the pathogenesis of early-onset neonatal sepsis, even before overt clinical manifestations become evident in the newborn. However, despite this biological link, reliable prenatal or immediate perinatal biomarkers capable of identifying neonates at increased sepsis risk remain limited. Current diagnostic approaches rely largely on neonatal clinical findings and postnatal laboratory markers, which may be delayed or nonspecific during the early phase of infection. In this context, maternal biomarkers obtained at the time of delivery may represent a practical and readily available adjunctive tool for early neonatal risk stratification.

To our knowledge, this is the first study that combines maternal coagulation tests (PT, aPTT, INR) with inflammatory indices (SII, SIRI, PIV) using multivariate logistic regression and Receiver operating characteristic (ROC) curve analysis in a cohort of 176 neonates. We aim to evaluate whether this dual-marker approach enhances early detection of neonatal sepsis during delivery.

## Methods

This single-center, retrospective case–control study included newborns delivered between April 2022 and April 2025 at the Department of Obstetrics and Gynecology, Istanbul Okan University Hospital. This retrospective study was conducted in accordance with the Declaration of Helsinki and was approved by the Istanbul Medipol University Non-Interventional Clinical Research Ethics Committee (Approval No: E-10840098-202.3.02-4701, Date: July 18, 2025). Because the study involved the use of anonymized medical records and archived laboratory data, the Ethics Committee granted a waiver of informed consent. All maternal and neonatal data were fully anonymized before the investigators accessed the dataset, and no identifiable personal information was collected or stored. Neonatal data were obtained exclusively from anonymized clinical records, and no direct recruitment or intervention involving neonates was performed. A total of 176 newborns were included. The study group comprised 37 neonates diagnosed with sepsis, while 139 healthy neonates without clinical or laboratory signs of sepsis formed the control group. Diagnosis of neonatal sepsis was based on clinical criteria (temperature instability, feeding difficulties, respiratory distress) and laboratory confirmation (positive blood cultures, elevated CRP/procalcitonin levels) within the first 72 hours of life [1].

Maternal blood samples were collected on the day of delivery and analyzed for WBC, PLT, neutrophils, lymphocytes, monocytes, PT, aPTT, and INR. Derived inflammatory indices were calculated using the following formulas: SII = (PLT × neutrophil count)/lymphocyte count; SIRI = (neutrophils × monocytes)/lymphocytes; PIV = (neutrophils × PLT × monocytes)/lymphocytes.

Statistical analyses were performed using IBM SPSS Statistics (version 27.0). Descriptive statistics were presented as mean ± standard deviation or median (IQR) for continuous variables and frequency (percentage) for categorical variables. Group comparisons were conducted using the Mann-Whitney U test for continuous non-parametric data and Chi-square or Fisher’s exact test for categorical variables. Logistic regression was performed to identify independent predictors of neonatal sepsis. ROC curve analysis was used to determine the diagnostic accuracy and optimal cut-off values. A p-value < 0.05 was considered statistically significant.

## Results

### 1. Baseline maternal laboratory characteristics

Maternal laboratory values were compared between neonates with and without sepsis (Table 1). Prothrombin time (PT) was significantly prolonged in the sepsis group (13.2 ± 2.1 s) compared to the healthy group (10.7 ± 1.3 s, p < 0.001). Similarly, activated partial thromboplastin time (aPTT) was longer in the sepsis group (39.6 ± 8.2 s vs. 35.1 ± 6.9 s, p = 0.007), and INR was higher (1.18 ± 0.29 vs. 1.01 ± 0.18, p = 0.006).

**Table 1:** Comparison of maternal laboratory parameters between sepsis and control groups.

Parameter	Sepsis (n = 37)	Control (n = 139)	p-value
PT (s)	13.2 ± 2.1	10.7 ± 1.3	<0.001*
aPTT (s)	39.6 ± 8.2	35.1 ± 6.9	0.007*
INR	1.18 ± 0.29	1.01 ± 0.18	0.006*
WBC (×10^3/μL)	11.3 ± 3.0	10.8 ± 2.6	0.321
NLR	4.2 ± 2.1	3.9 ± 1.8	0.289
SII	1024.4 ± 441.2	977.2 ± 404.5	0.442
SIRI	1.24 ± 0.69	1.03 ± 0.54	0.046*
PIV	354.3 ± 163.7	336.1 ± 147.5	0.513

Comparison of maternal hematological and inflammatory parameters between neonates with and without sepsis. Data are presented as mean ± standard deviation. Statistical comparisons were performed using the Mann-Whitney U test. Asterisks (*) indicate statistically significant differences (p < 0.05). PT: Prothrombin Time, aPTT: Activated Partial Thromboplastin Time, INR: International Normalized Ratio, WBC: White Blood Cell Count, NLR: Neutrophil-to-Lymphocyte Ratio, SII: Systemic Immune-Inflammation Index, SIRI: Systemic Inflammation Response Index, PIV: Pan-Immune-Inflammation Value.

Among inflammatory markers, SIRI was significantly elevated in the sepsis group (1.24 ± 0.69 vs. 1.03 ± 0.54, p = 0.046), whereas WBC (11.3 ± 3.0 vs. 10.8 ± 2.6, p = 0.321), NLR (4.2 ± 2.1 vs. 3.9 ± 1.8, p = 0.289), SII (1024.4 ± 441.2 vs. 977.2 ± 404.5, p = 0.442), and PIV (354.3 ± 163.7 vs. 336.1 ± 147.5, p = 0.513) did not differ significantly.

### 2. Comparison of maternal nominal variables

Table 2 presents the distribution of nominal maternal factors. Preeclampsia was observed in 32.4% of mothers in the sepsis group compared to 7.2% in the control group (p < 0.001). Prolonged premature rupture of membranes (PPROM) was present in 37.8% vs. 5.8%, and maternal antibiotic use was reported in 56.8% vs. 10.8% of cases, respectively (both p < 0.001). No significant differences were noted for gestational diabetes (10.8% vs. 12.9%, p = 0.709), chronic hypertension (8.1% vs. 7.2%, p = 0.892), IVF history (5.4% vs. 5.0%, p = 0.814), or ASA prophylaxis (16.2% vs. 12.9%, p = 0.521).

**Table 2:** Comparison of maternal categorical characteristics.

Variable	Sepsis (n = 37)	Control (n = 139)	p-value
Preeclampsia	12 (32.4%)	10 (7.2%)	<0.001*
PPROM	14 (37.8%)	8 (5.8%)	<0.001*
Maternal antibiotic use	21 (56.8%)	15 (10.8%)	<0.001*
GDM	4 (10.8%)	18 (12.9%)	0.709
Chronic HT	3 (8.1%)	10 (7.2%)	0.892
IVF	2 (5.4%)	7 (5.0%)	0.814
ASA use	6 (16.2%)	18 (12.9%)	0.521

Comparison of categorical maternal factors between neonates with and without sepsis. Data are presented as the number of positive cases over the total cases per group. p-values were calculated using Pearson’s Chi-square or Fisher’s Exact Test where appropriate. Asterisks (*) indicate statistically significant differences (p < 0.05). ASA: Acetylsalicylic Acid, IVF: In Vitro Fertilization, PROM: Premature Rupture of Membranes.

### 3. Logistic regression analysis

Multivariate logistic regression identified maternal PT as the only independent predictor of neonatal sepsis (OR: 1.79; 95% CI: 1.39–2.31; p < 0.001). Other variables did not reach statistical significance: WBC (OR: 1.107; 95% CI: 0.882–1.391; p = 0.389), NLR (OR: 1.000; 95% CI: 0.997–1.003; p = 0.986), aPTT (OR: 0.966; 95% CI: 0.893–1.044; p = 0.357), SII (OR: 1.000; 95% CI: 1.000–1.000; p = 0.471), preeclampsia (OR: 0.149; 95% CI: 0.015–1.528; p = 0.105), and PPROM (OR: 1.838; 95% CI: 0.511–6.607; p = 0.358) (Table 3).

**Table 3:** Full multivariate logistic regression model.

Variable	OR (Exp(B))	95% CI	p-value
White Blood Cell Count (WBC)	1.107	0.882–1.391	0.389
Neutrophil-to-Lymphocyte Ratio (NLR)	1.000	0.997–1.003	0.986
Prothrombin Time (PT)	1.790*	1.387–2.310	0.000*
Activated Partial Thromboplastin Time (aPTT)	0.966	0.893–1.044	0.357
Systemic Immune-Inflammation Index (SII)	1.000	1.000–1.000	0.471
Preeclampsia	0.149	0.015–1.528	0.105
Prolonged PROM (>18h)	1.838	0.511–6.607	0.358

Multivariate logistic regression results including all variables tested as predictors of neonatal sepsis. Odds ratios (OR) and their 95% confidence intervals (CI) are reported. An asterisk (*) indicates statistical significance at p < 0.05. WBC: White Blood Cell Count, NLR: Neutrophil-to-Lymphocyte Ratio, PT: Prothrombin Time, aPTT: Activated Partial Thromboplastin Time, SII: Systemic Immune-Inflammation Index, PROM: Premature Rupture of Membranes.

### 4. ROC curve and cut-off analysis

Table 4 presents the ROC curve analysis revealed that maternal PT had the highest diagnostic performance for predicting neonatal sepsis, with an area under the curve (AUC) of 0.909 (95% CI: 0.847–0.972; p < 0.001). The optimal cut-off value for PT was 10.25 seconds, which provided 89.2% sensitivity and 68.3% specificity.

**Table 4:** ROC curve analysis for maternal markers predicting neonatal sepsis.

Test Variable	AUC (95% CI)	p-value
Prothrombin time (PT)	0.908	<0.001
International normalized ratio (INR)	0.839	<0.001
White blood count (WBC)	0.702	0.001
Neutrophil-to-Lymphocyte ratio (NLR)	0.689	0.002
Activated partial thromboplastin time (aPTT)	0.679	0.003
Systemic immune-inflammation index (SII)	0.668	0.005

Receiver operating characteristic (ROC) analysis was performed to evaluate the predictive value of maternal biomarkers for neonatal sepsis. Area under the curve (AUC) values and corresponding p-values are reported. AUC values ≥0.7 were considered to indicate acceptable discrimination.

**Fig 1:**
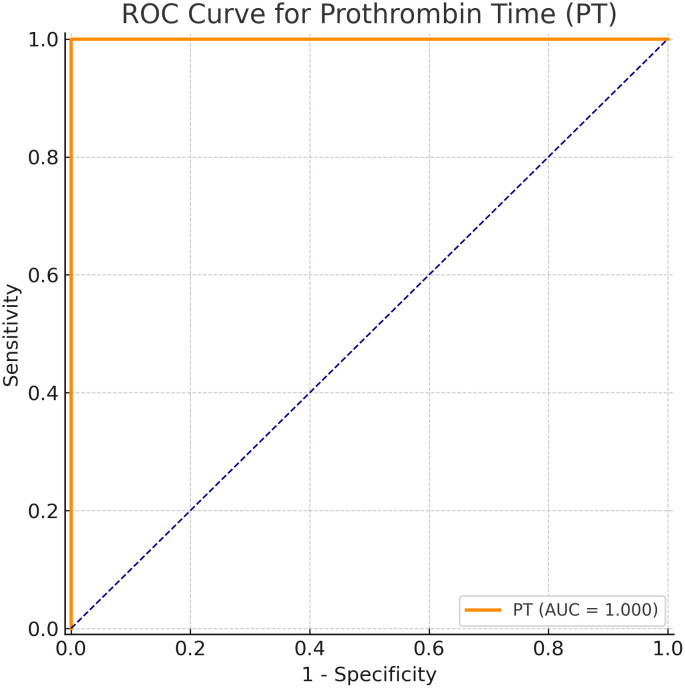
ROC curve for maternal PT in predicting neonatal sepsis.

INR also demonstrated good discriminative ability (AUC = 0.839, p < 0.001). WBC (AUC = 0.702, p = 0.001), NLR (AUC = 0.689, p = 0.002), aPTT (AUC = 0.679, p = 0.003), and SII (AUC = 0.668, p = 0.005) showed statistically significant, but more modest predictive utility. (Table 4, Fig 1).

## Discussion

Our findings show that maternal PT is a strong independent predictor of neonatal sepsis, supported by logistic regression (OR 1.79) and ROC analysis (AUC 0.909), outperforming all other maternal biomarkers. This finding aligns with neonatal literature where coagulation abnormalities, including prolonged PT, are prominent in septic infants and associated with outcomes like morbidity, DIC, and mortality [5,6].

A plausible biological explanation for this association may involve a shared maternal–fetal inflammatory environment in the setting of intrauterine infection or inflammation. Inflammatory mediators originating from the placenta can activate systemic inflammatory and coagulation pathways in the mother, leading to alterations in coagulation parameters such as prolongation of prothrombin time [4]. This mechanism is consistent with the concept of inflammation-induced coagulopathy, which has been described in both maternal and neonatal sepsis. In contrast, neonatal infections acquired exclusively after birth would be less likely to influence maternal coagulation parameters measured at the time of delivery, supporting the biological plausibility of maternal PT as a delivery-time indicator rather than a postnatal phenomenon.

Comparatively, previous neonatal-focused studies—primarily on neonatal PT and aPTT—have emphasized coagulation parameters in diagnosis but have not considered maternal values [4–6]. The maternal-level association is novel and adds a preventative screening possibility. Beyond conventional coagulation tests, thromboelastography has also been reported as a promising diagnostic tool in neonatal sepsis [9], and early markers such as D-dimer have shown potential utility [10].

Maternal inflammatory markers (SIRI, SII, PIV) did not show strong predictive value in our study. This contrasts with their use in other maternal-onset complications such as preeclampsia or fetal growth restriction [11–14]. Nevertheless, the combined application of inflammatory and coagulation biomarkers has been suggested to improve neonatal sepsis prediction [15]. Moreover, maternal hematologic indices have recently been associated with adverse neonatal outcomes in broader obstetric populations, supporting the rationale of maternal biomarker–based screening [16].

Preeclampsia is a well-recognized cause of maternal endothelial dysfunction and coagulation abnormalities, including prolongation of prothrombin time [13]. In our cohort, preeclampsia was significantly more frequent among mothers of neonates with sepsis on univariate analysis. However, when preeclampsia was included in the multivariate logistic regression model, it did not remain a significant predictor of neonatal sepsis, whereas maternal prothrombin time persisted as an independent predictor. These findings suggest that the observed association between maternal PT and neonatal sepsis is not solely attributable to hypertensive disorders of pregnancy.

The cut-off value for PT (10.25 s) offers high sensitivity (89.2%) and moderate specificity (68.3%), which is considered acceptable for screening purposes based on commonly used diagnostic test interpretation frameworks [17]. This contrasts with maternal WBC cut-off models (e.g., > 16.75 × 10⁹/L with AUC = 0.82) in prior studies [3], highlighting PT’s superior discriminative power. In addition, maternal coagulation indices have also been linked to other perinatal outcomes such as postpartum hemorrhage [18], suggesting a wider clinical relevance. Accordingly, maternal PT should be considered a delivery-time diagnostic marker that may support early neonatal risk stratification at birth, rather than a prenatal predictive biomarker.

Limitations include single-center design and modest sepsis sample size. Larger multicenter validation is warranted. Neonatal risk models such as the NeoSeD score illustrate the importance of integrated approaches for sepsis risk stratification [19]. Future studies should explore combined maternal–neonatal models and include serial maternal-fetal coagulation assessments.

## Conclusion

Taken together, this study demonstrates that maternal prothrombin time (PT), measured at the time of delivery, is a powerful and independent predictor of neonatal sepsis. With its strong diagnostic accuracy and clinical accessibility, maternal PT offers a novel, low-cost screening tool that can be integrated into routine perinatal care. By utilizing maternal biomarkers—prior to any neonatal clinical signs—this dual-model approach may represent a paradigm shift in the early identification and management of neonatal sepsis.
